# Why do they sign but not consult? A study on the effectiveness of China's family doctor contract services from the perspective of patient-doctor interaction

**DOI:** 10.3389/fmed.2026.1848658

**Published:** 2026-08-04

**Authors:** Xiangfei Li, Yuxin Shi, Xin Li

**Affiliations:** 1 School of Economics and Management, Tiangong University, Tianjin, China; 2 Beijing Tianjin Hebei Health Collaborative Development Research Center, Tiangong University, Tianjin, China; 3 College of Management and Economics, Tianjin University, Tianjin, China; 4 Tianjin Women and Children's Health Center, Tianjin, China

**Keywords:** family doctor contract services, health services utilization, hierarchical medical system, patient-centered care, perceived quality, primary care

## Abstract

**Background:**

Family doctor contract services (FDCS) in China aim to promote primary care first-contact, yet the “sign but no consult” phenomenon persists Traditional accessibility models fail to explain the heterogeneity in patient behavior. This study introduces Interactive Interpersonal Perceived Quality (IIPQ) as a key construct to understand why and for whom FDCS is effective, from a person-centered perspective.

**Methods:**

We conducted a cross-sectional study using data from the 2024 Tianjin family doctor service resident survey, which included 4,578 residents across 16 districts in Tianjin, China. We measured potential accessibility (geographic, financial), realized accessibility, and IIPQ (comprising perceived feasibility, desirability, and continuity). The primary outcome was Family Doctor First-Contact Behavior (FDFCB). Latent Profile Analysis (LPA) was employed to identify distinct patient subgroups based on their IIPQ scores, followed by logistic regression to examine the moderating effects.

**Results:**

Three distinct patient profiles were identified via LPA: a low perceived quality group, a moderate perceived quality group and a high perceived quality group. Realized accessibility positively predicted FDFCB, and its effect was significantly moderated by IIPQ, with perceived continuity playing a key role. Notably, traditional potential accessibility measures (geographic and financial) showed no significant direct effect on FDFCB after accounting for other factors.

**Conclusion:**

Patients are not a homogenous group; their subjective perception of the doctor-patient relationship is a critical, and often overlooked, determinant of FDCS effectiveness. The impact of service accessibility depends on patients' interpersonal experience. The true value of FDCS lies in its capacity to shift patients' decision-making logic from a reliance on structural convenience to a reliance on relational trusts.

## 1 Introduction

The contradiction between insufficient medical resources and residents' demand for medical services has always been a major challenge facing China. To enable people to fully access healthcare services and alleviate pressure on large hospitals, the Chinese government has comprehensively implemented the family doctor system since 2016. The policy aims to encourage patients to seek initial treatment at the grassroots level in the hope that the contract mechanism will alleviate the pressure on tertiary hospitals (1).

The family doctor contracted service system is not an isolated policy initiative, but a concentrated embodiment of the long-term evolution of China's primary healthcare (PHC) policy system. Since the launch of the new healthcare reform in 2009, China has continuously promoted the construction of the primary medical care system centered on community health services. In 2015, the State Council issued the hierarchical medical treatment system, alleviating the difficulties in accessing medical resources. The full-scale rollout of family doctor contract services (FDCS) in 2016 marked the ultimate foothold of the above policy evolution path, forming the core organizational model linking residents and primary medical institutions within China's PHC system.

The FDCS is essentially a health-management agreement mechanism centered on the family doctor as the responsible party: residents voluntarily enter into a long-term, stable contractual relationship with community health service institutions, under which family doctor teams assume responsibility for continuous services including health assessment, chronic disease management, and referral coordination. This mechanism entitles contracted residents to a personalized health management plan at the grassroots level—typically encompassing health education, regular follow-up, medication guidance, and bidirectional referral arrangements, dynamically adjusted according to the resident's evolving health status. International evidence suggests that the effectiveness of such relationship-based, contractual health management mechanisms depends substantially on the stability and trust embedded in the interaction between service providers and residents (2, 3).

However, this intended effect of institutional design has not been fully realized in practice. In recent years, as policies have been continuously strengthened, the coverage rate of family doctor contracts has steadily increased. However, the phenomenon of “sign but not consult” has become increasingly prominent in reality (4–6). Although a large number of residents have signed up for family doctor services, many patients with chronic and non-communicable diseases still bypass family doctors and seek treatment at large hospitals when they need medical care, rendering the gatekeeper function of the family doctor contract system severely ineffective (7, 8).

This phenomenon reveals the limitations of traditional research paradigms. Existing studies have primarily focused on structural factors on the supply side, such as optimizing resource allocation after increasing resource input. In contrast, on the demand side, they tend to simplify patients into a group that responds homogeneously to single indicators, such as satisfaction and trust (9–11). This perspective overlooks the essence of patients' healthcare decision-making as a complex psychological process, particularly failing to capture the critical variable of interpersonal quality in doctor-patient interactions, such as communication methods and emotional care (12, 13). When patients cannot establish positive emotional connections and trust during interactions, relying solely on physical convenience is insufficient to retain them (14, 15).

This study takes Tianjin—China's first city to legislate family doctor contract services—as a case study, aiming to analyse and resolve the current widespread “sign but not consult” dilemma from the perspective of doctor-patient interaction perception. The core contribution of this study lies in shifting the research focus from universal objective policy measures and service supply to capturing patients' key subjective experiences through Interactive Interpersonal Perceived Quality (IIPQ); furthermore, we employ latent profile analysis (LPA), a human-centered research method, to reveal how patient subgroups with different perception patterns exhibit differentiated behavioral responses to contract services.

## 2 Literature review and hypothesis development

### 2.1 The impact of potential accessibility on patients' family doctor first-contact behavior

In classical theories of health services research, potential accessibility is regarded as a foundational hardware condition influencing residents' healthcare-seeking behavior. It is defined as the objective possibility for individuals to access healthcare services (16), primarily composed of two core dimensions: geographic accessibility and economic affordability (17, 18). Extensive empirical research has confirmed that removing these structural barriers is a necessary pre-requisite for guiding residents to utilize family doctor services (19).

Based on this theoretical and empirical foundation, this study first proposes the following benchmark research hypothesis:

H1: potential accessibility (including geographical and financial dimensions) has a direct positive impact on patients' family doctor first-contact behavior.

### 2.2 The impact of realized accessibility on patients' famliy doctor first-contact behavior

In China's latest healthcare reform, despite the proximity of many primary healthcare institutions and substantial investments in upgrading their facilities (20, 21), or through policy measures to systematically reduce service utilization costs (22), residents still choose to seek care elsewhere, bypassing primary healthcare institutions.

This phenomenon indicates that once basic structural pre-requisites are fulfilled, relying exclusively on policy-level factors is inadequate to explain the complexities of patient decision-making, thereby revealing the limitations of a unidimensional perspective. Consequently, it is essential to investigate the primary determinants that drive patient adoption and sustained engagement after initial access barriers have been overcome.

To address the explanatory challenges of potential accessibility theory, this study further introduces the concept of realized accessibility (14). It shifts the focus of research from objective possibility to subjective fit, emphasizing the intrinsic quality of service provision. Among the various conceptual frameworks developed in accessibility research, the five-dimensional model proposed by Penchansky and Thomas remains one of the most widely applied and theoretically mature frameworks. Its core strength lies in moving beyond a narrow focus on the mere existence of resources, instead defining accessibility in terms of the degree of fit between service provision and patient needs—making it particularly well-suited to explaining why residents may still bypass primary care even after structural barriers have been removed (23). However, as this model was originally developed within the context of conventional Western healthcare services, the conceptual content and relative weighting of its dimensions may not fully correspond to the institutional characteristics of China's family doctor contract service. Considering that China's family doctor contract service differs fundamentally from traditional medical services, it is a long-term health management model based on a relationship of trust (24, 25). For this reason, this study draws on the classic accessibility model proposed by Penchansky & Thomas and adapts it to the Chinese context. The model is reconfigured into four dimensions:

Availability measures whether the quantity and type of service resources are sufficient, such as relevant facilities, equipment, medications, and service providers (26, 27).

Acceptability measures the quality of the healthcare environment and the level of respect shown to patients, which form the foundation of a positive healthcare experience (28).

Accommodation refers to the convenience and flexibility of service processes, measuring whether the design of processes such as appointments, waiting times, and referrals is patient-centered and adaptable to the habits and needs of different patient groups (23).

Accountability, a newly added dimension based on China's policy context, refers to the institutionalized commitment of family doctors to assume ongoing responsibility in the course of service delivery, including proactive follow-up, responsiveness to patient needs, and the maintenance of continuity of patient information (24, 25).

Therefore, based on the above analysis, we propose the following research hypotheses:

H2: realized accessibility has a direct positive impact on patients' family doctor first-contact behavior.

### 2.3 Interactive interpersonal perceived quality: the core variable reshaping the doctor-patient relationship

Even if the family doctor service system achieves extremely high standards in all aspects of service provision, its ultimate value must be conveyed and realized through the interactive process between doctors and patients. This interactive process is the critical moment when the objective level of service provision transitions to the patient's experience and is also the decisive moment when patients form their final perceptions (29).

**Figure 1:**
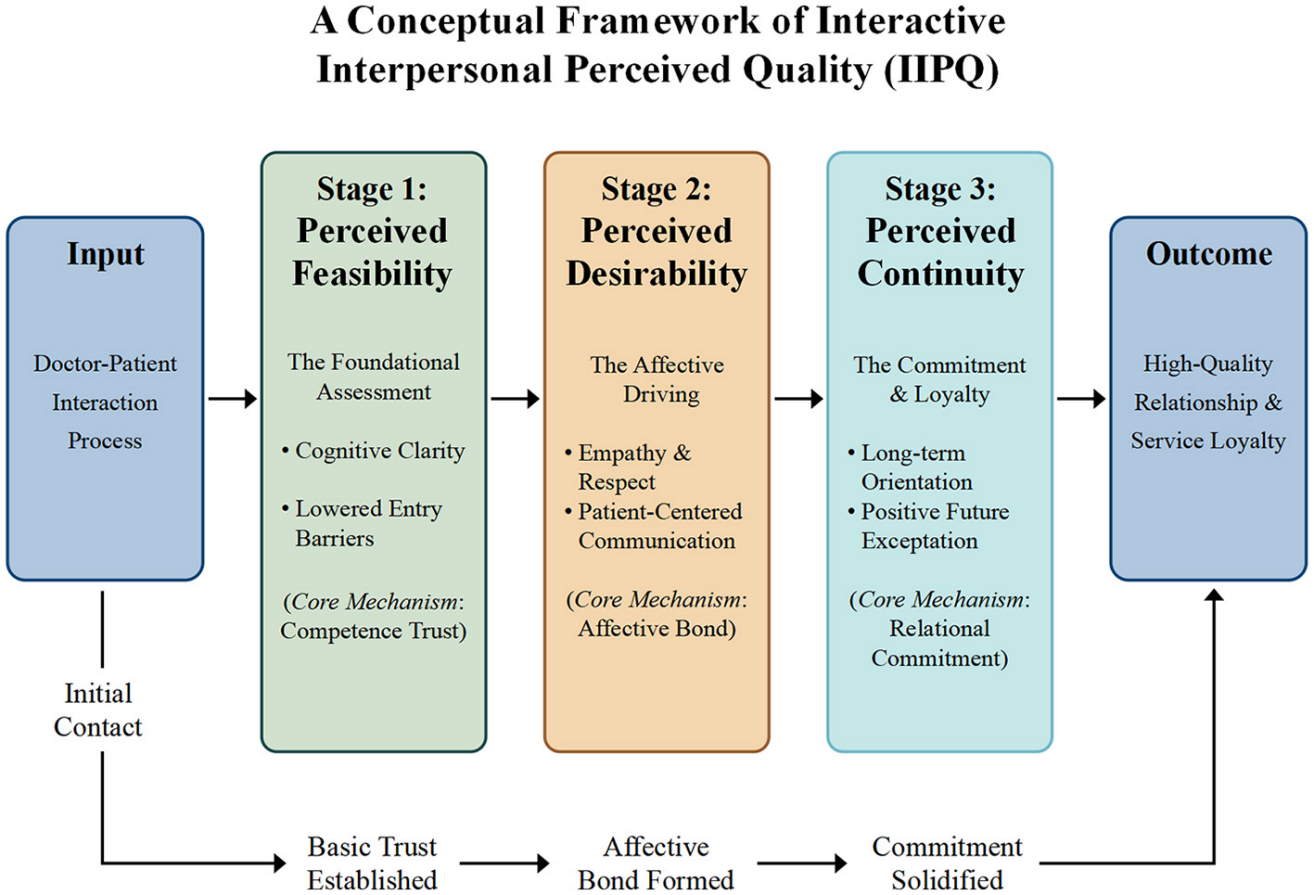
A conceptual framework of interactive interpersonal perceived quality. The framework illustrates how the Doctor-Patient Interaction Process (Input) translates into tangible outcomes through three progressive stages. The three stages of interactive interpersonal perceived quality are: perceived feasibility, perceived desirability and perceived continuity.

To address this, this study introduces the core variable of IIPQ, defined as patients' comprehensive and subjective evaluation of the interpersonal interaction process during direct interactions with service providers. This concept inherently encompasses three progressively related dimensions, as shown in Figure 1, reflecting the perceptual pathway from initial contact to relationship establishment and ultimately to loyalty maintenance, aligning with the stage-based characteristics of the doctor-patient relationship development (30).

This conceptualization is also consistent with the broader literature on Patient-Reported Experience Measures (PREMs) and the OECD PaRIS conceptual framework, which emphasize patients' own reports of how they experience care, including domains such as accessibility, care coordination, and provider-patient communication (31). Within this broader patient-experience framework, realized accessibility and IIPQ capture different but related domains. Realized accessibility reflects the practical and service-delivery layer of experience, namely whether family doctor services are available, acceptable, accommodating, and accountable in meeting patients' needs. By contrast, IIPQ focuses on the relational and interpersonal layer of experience, namely whether patients perceive interactions with family doctors as feasible, trustworthy, respectful, emotionally supportive, and worth maintaining over time. Therefore, although both constructs are measured from the patient perspective, realized accessibility mainly captures the functional fit between service provision and patient needs, whereas IIPQ captures the quality of the doctor-patient relationship formed through interaction.

Herzberg's two-factor theory, originally developed in the context of organizational behavior and employee motivation, posits that the factors influencing satisfaction and dissatisfaction operate through two qualitatively distinct mechanisms rather than along a single continuum (32). Hygiene factors—such as working conditions, policies, and interpersonal relations—do not, by themselves, generate satisfaction; however, their absence or inadequacy produces dissatisfaction. In contrast, motivators—such as recognition, responsibility, and achievement—are the factors capable of generating genuine satisfaction and engagement, though their absence does not necessarily lead to dissatisfaction. This conceptual distinction has since been extended beyond organizational settings to service quality and patient experience research, where it has proven useful in explaining why merely meeting baseline service expectations is insufficient to generate positive relational outcomes, while addressing higher-order relational needs can meaningfully deepen patient engagement.

Perceived feasibility is the foundational assessment stage for relationship establishment. During this stage, the patient's core task is to evaluate, through interaction, whether the service is relatively easy to understand and use (33). This dimension focuses on reducing patients' psychological and procedural entry barriers, similar to the hygiene factors in Herzberg's two-factor theory. The absence of this dimension directly leads to relationship breakdown, but its mere satisfaction is insufficient to drive relationship deepening.

Perceived Desirability is the emotional driving stage of relationship deepening. After feasibility is satisfied, patients shift their assessment to the humanistic care in the interaction process, i.e., whether they feel respected, empathized with, and involved in decision-making (34). This corresponds to the motivational factors in the two-factor theory and is the core driving force that propels patients from functional dependence to emotional preference.

Perceived Continuity is the commitment maintenance stage of relationship loyalty. At this stage, patients assess whether this high-quality relationship is worth maintaining in the long term (35). It reflects patients' positive expectations based on past interactions and their willingness to maintain a long-term relationship, with the core being the establishment of mutual commitment in the relationship.

The doctor-patient interaction process is a complex socio-psychological process that profoundly influences patients' satisfaction, compliance, and even health outcomes, making it an important factor influencing residents' family doctor first-contact behavior (FDFCB) (36). The IIPQ system systematically captures the quality of this core interaction process, and we have sufficient theoretical basis

Therefore, based on the above analysis, we propose the following hypothesis:

H3: interactive interpersonal perceived quality has a direct positive impact on patients' family doctor first-contact behavior.

### 2.4 The moderating effect of interactive interpersonal perceived quality from a population heterogeneity perspective

In the practice of the family doctor contract service system, IIPQ is not an objective and constant evaluation but exhibits significant individual heterogeneity. Previous studies have confirmed that factors such as patients' age, type of illness, duration of illness, and self-rated health status significantly influence their subjective perceptions of communication quality and trust during doctor-patient interactions (37–39). This suggests that treating all patients as a homogeneous group may obscure the complex mechanisms underlying service outcomes (40).

Subgroups with different IIPQ patterns will inevitably exhibit distinct behavioral response pathways under the same accessibility conditions. In fact, the role of perception quality or similar subjective experience variables as moderators in regulating their significant influence on behavioral pathways has been validated in multiple fields such as smart education and sustainable consumption (41, 42). It is reasonable to infer that this moderating effect also exists—and may even be more significant—in the relationship-oriented family doctor service domain (43).

Therefore, based on the above analysis, we propose the following research hypothesis:

H4: residents' interactive interpersonal perceived quality can be divided into different meaningful groups.

H5: the impact of realized accessibility on patients' family doctor first-contact behavior varies across different groups of perceived quality.

**Figure 2:**
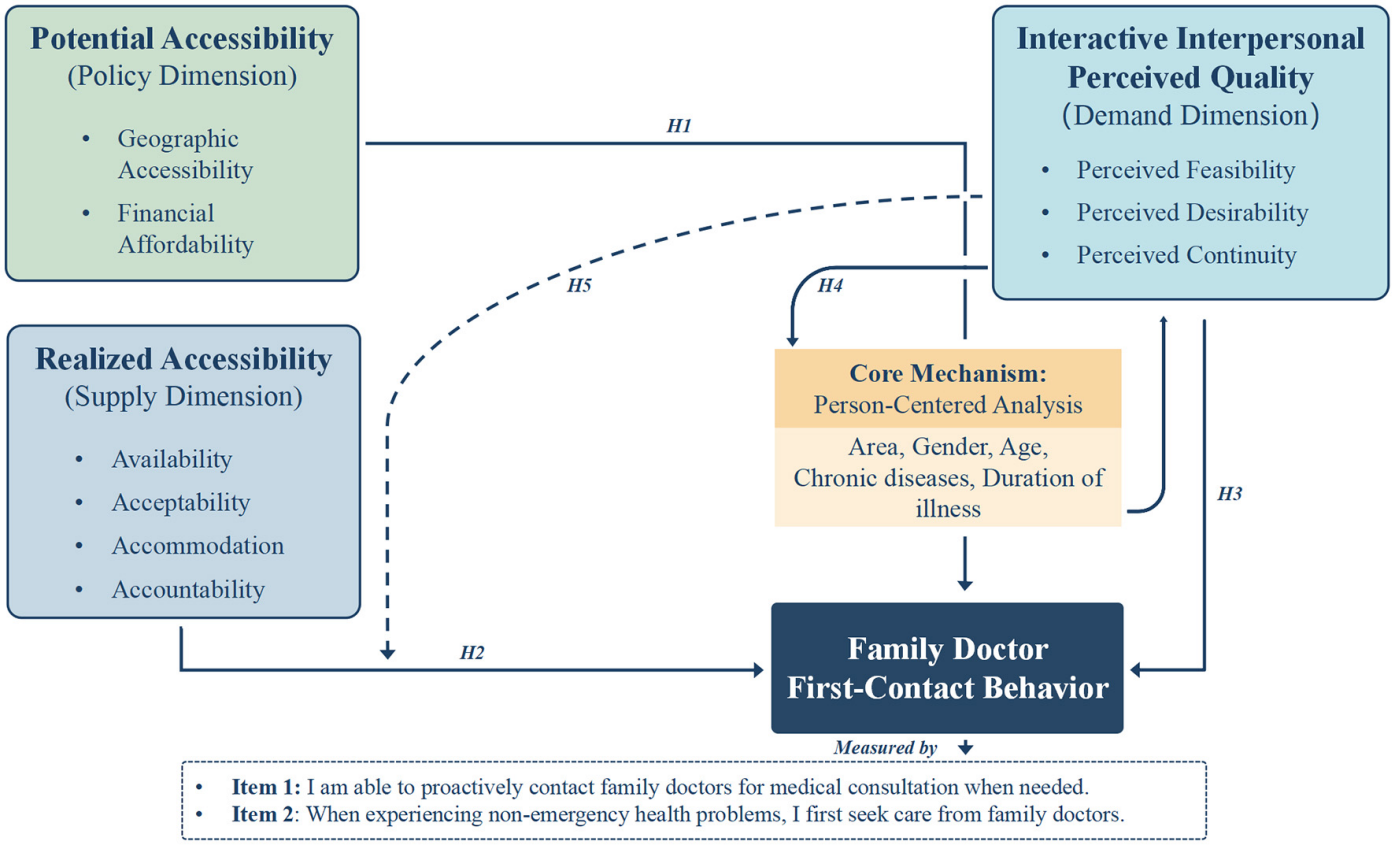
The hypothetical model of the study. The model illustrates the hypothesized pathways influencing Family Doctor First-Contact Behavior (FDFCB). It posits that Potential Accessibility (Policy Dimension), Realized Accessibility (Supply Dimension), and Interactive Interpersonal Perceived Quality (Demand Dimension) all have direct effects on FDFCB (H1, H2, H3). The Core Mechanism block signifies that the study employs a person-centered approach (Latent Profile Analysis) to identify patient subgroups based on their IIPQ, which is influenced by demographic and health characteristics (H4). The dashed arrow (H5) represents the central hypothesis of a moderating effect, suggesting that the relationship between Realized Accessibility and Family Doctor First-Contact Behavior is moderated by the patient's IIPQ profile.

The full conceptual framework integrating all of the above hypotheses is presented in Figure 2.

## 3 Materials and methods

### 3.1 Study area

Tianjin is an important municipality in northern China with a permanent resident population exceeding 13 million. Against the backdrop of national healthcare reform and the development of a tiered healthcare delivery system, Tianjin has been involved in related pilot initiatives since 2015 and has progressively advanced the establishment of an integrated, primary care–based service delivery system. In 2017, Tianjin fully implemented the family doctor contract service policy across the city, continuously strengthening mechanisms for care continuity and coordinated service delivery at the primary care level. In December 2022, Tianjin issued local regulations on family doctor contract services, formally institutionalizing the service model and providing regulatory support for its long-term implementation.

The municipality consists of 16 districts, including six highly urbanized central districts (Heping, Nankai, Hexi, Hedong, Hebei, and Hongqiao) and ten surrounding districts characterized by mixed urban–rural contexts. These districts exhibit substantial heterogeneity in population density, healthcare resource allocation, and patterns of healthcare utilization, thereby providing a structurally diverse setting suitable for empirical analysis across different population groups.

**Figure 3:**
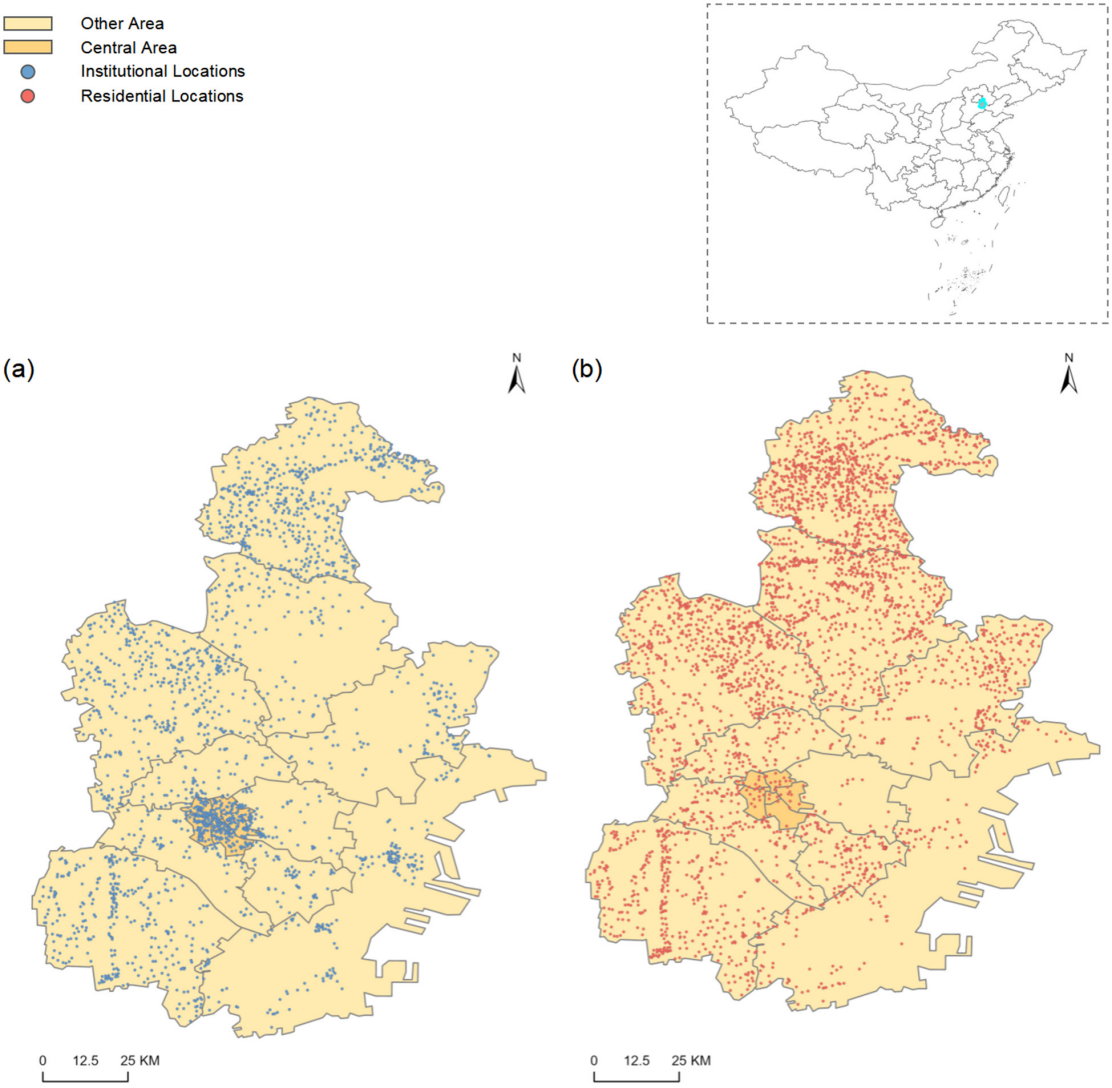
Spatial distribution of primary care institutions and sample residential locations in the study area of Tianjin, China. The figure illustrates the geographic context of the study. The inset map (top right) indicates the location of Tianjin municipality within China. The main maps display the administrative boundaries of Tianjin, distinguishing between the six central urban districts (Central Area, orange) and the surrounding districts (Other Area, yellow). **(a)** shows the spatial distribution of the primary care institutional locations (blue dots) included in the study. **(b)** shows the spatial distribution of the residential locations (red dots) of the surveyed participants.

Tianjin was selected as the study area because it provides a relatively mature and policy-relevant setting for the implementation of family doctor contract services, with a clear policy evolution pathway and stable operational environment. At the same time, its pronounced intra-city heterogeneity enhances its suitability for examining patients' perceptions of family doctor services. Figure 3 presents the administrative divisions of Tianjin, along with the spatial distribution of family doctor contract service institutions and population distribution.

### 3.2 Data collection and collation

A stratified cluster sampling strategy was used to conduct the field survey. First, the 16 administrative districts of Tianjin were used as the primary strata. Considering differences in population size, urban–rural characteristics, and the distribution of primary healthcare resources across districts, primary healthcare institutions were selected within each district as survey sites. In total, 267 primary healthcare institutions were included, covering all 16 districts of Tianjin, thereby ensuring regional coverage and structural heterogeneity of the sample.

For patient recruitment, patients who visited the selected primary healthcare institutions on the survey day were used as the sampling population. Approximately 5% of the patients visiting each institution on the survey day were selected using simple random sampling. Trained investigators distributed the questionnaires and provided standardized explanations on site. Before the survey, participants were informed of the study purpose, survey content, anonymity of responses, and the voluntary nature of participation. The questionnaire survey was conducted after informed consent was obtained. A total of 4,578 patients were ultimately included in the study sample.

The inclusion criteria were as follows: (1) having basic written or verbal communication ability; (2) being able to accurately understand the questionnaire and complete it independently or with necessary clarification from the investigator; and (3) being informed of the study purpose and willing to participate in the survey. The exclusion criteria were as follows: having mental disorders, severe communication difficulties, hearing impairment, or physical conditions that prevented completion of the questionnaire.

### 3.3 Dependent variable: family doctor first-contact behavior

In this study, “family doctor” refers to physicians providing family doctor services in primary care settings. FDFCB reflects residents' perceived tendency to regard family doctors as the first point of contact for non-emergency healthcare needs. FDFCB was measured using two items: “I am able to proactively contact family doctors for medical consultation when needed” and “When experiencing non-emergency health problems, I first seek care from family doctors.” The complete questionnaire is provided in the Supplementary materials. Each item was scored on a 1–5 scale, with higher scores indicating a stronger perceived orientation toward family doctors as first-contact providers. The scores of the two items were summed to generate the total FDFCB score, which ranged from 2 to 10. Before analysis, this summation was performed to construct the FDFCB variable.

### 3.4 Measurement

Potential accessibility (geographic accessibility and financial affordability) was measured using two single-item measures. Realized accessibility in terms of service supply was measured using four dimensions. IIPQ, considered from the patient's perspective, comprises three progressive dimensions, capturing the core interpersonal perceptions during doctor-patient interactions. Except for geographic accessibility, all other items were scored using a 5-point Likert scale. The detailed measurement items and their sources are presented in Table 1.

**Table 1:** Measurement scale.

Attributes	Measurement
Potential accessibility dimension	
Geographic accessibility	Is it convenient for you to walk from your home to the nearest community health service center (or a primary care institution providing family doctor services; approximately within 15 min)?
Financial affordability	Do you consider the current family doctor contract services to be affordable for you?
Realized accessibility dimension	
Availability	The services provided by family doctors generally meet my health needs.
	The facilities provided by family doctors are sufficient to meet my needs.
	There are sufficient numbers of family doctors available in the community to provide family doctor services.
	Items derived from PAHCQ (56).
Acceptability	Family doctors are neatly dressed and professional.
	The clinic environment for family doctor services is comfortable, clean, and convenient.
	Family doctors can accurately record my information and maintain confidentiality.
	Items derived from PCAT-C (57).
Accommodation	The process of receiving family doctor services is simple and efficient.
	Family doctors offer multiple communication methods (e.g., phone, online consultation, etc.).
	Family doctors flexibly arrange appointment times according to my specific needs.
	Items derived from PAHCQ (56).
Accountability	Family doctors proactively follow up on my health status.
	If I have further needs, family doctors will help me coordinate referrals.
	Family doctors can remember my medical history and treatment arrangements.
	Items derived from PCAT-C (57).
Interactive interpersonal perceived quality dimension	
Perceived feasibility	I know how to contact family doctors.
	I believe that family doctor services are feasible under current conditions.
	I believe that family doctors can address my common minor health issues.
	Family doctor services are convenient for me to use.
	Items derived from Feasibility of Intervention Measure (FIM)(58).
Perceived desirability	I believe that the advice provided by family doctors is trustworthy.
	I feel that family doctors are extremely thorough and attentive.
	The services provided by family doctors make me feel respected and understood.
	I am willing to contact family doctors because I believe they will provide me with a comprehensive treatment plan.
	Items derived from Validation of the General Trust in Physicians Scale (35).
Perceived continuity	Family doctors are very familiar with my situation and that of my family members.
	I am willing to maintain a long-term relationship with family doctors.
	I have established a stable communication mechanism with family doctors.
	Items derived from the Chao PC scale Section 2 (59).
Family doctor first-contact behavior	
	I am able to proactively contact family doctors for medical consultation when needed.
	When experiencing non-emergency health problems, I first seek care from family doctors.

The table presents the measurement items used to operationalize the key theoretical constructs in this study. Potential Accessibility was measured using single items for each of its two dimensions. Realized Accessibility and Interactive Interpersonal Perceived Quality (IIPQ) were measured using multi-item scales. The source for each adapted or adopted scale is provided.

### 3.5 Data analysis

All statistical analyses were performed using IBM SPSS 26.0 and Mplus 8.3, with a significance level set at α = 0.05. The analysis proceeded in four main stages.

First, we assessed the psychometric properties of the main scales. Sampling adequacy and factorability were examined using the Kaiser-Meyer-Olkin (KMO) measure and Bartlett's test of sphericity. Exploratory factor analysis was conducted to examine the underlying factor structure. Internal consistency reliability was assessed using Cronbach's alpha, standardized alpha, corrected item-total correlations, and alpha-if-item-deleted statistics. Single-item dimensions were reported descriptively and were not assessed using Cronbach's alpha.

Second, descriptive statistics were calculated for all study variables.

Third, we employed LPA in Mplus to identify distinct subgroups of patients based on their Interactive Interpersonal Perceived Quality (IIPQ). The optimal number of profiles was determined by comparing model fit indices (AIC, BIC, aBIC), classification accuracy (Entropy), and likelihood ratio tests (LMR-LRT, BLRT), alongside theoretical interpretability.

Fourth, we conducted a series of regression analyses to test our hypotheses. Binary logistic regression was used to explore the factors predicting membership in the identified latent profiles. To test the main and moderating effects on the primary outcome, we used multiple linear regression and hierarchical logistic regression. This allowed us to first examine the direct effects of potential accessibility, realized accessibility, and IIPQ on Family Doctor First-Contact Behavior, and subsequently, to test the hypothesized moderating effect of the IIPQ latent profiles on the relationship between realized accessibility and the outcome.

## 4 Results

### 4.1 Descriptive statistics

As shown in Table 2, descriptive statistics for all categorical data are presented. Most participants resided outside the central area (82.5%), female (65.9%), under 45 years old (51%), without chronic diseases (68.5%) [among participants with chronic diseases, the duration of illness was concentrated between 5 and 10 years (72%)], found it convenient to reach the nearest community health service station (52.5%), and had a contracted family doctor (91.2%). Since all continuous variables were not normally distributed, the median and interquartile range were used to describe the distribution characteristics of the data, as shown in Table 3.

**Table 2:** Characteristics of participants (categorical variables).

Variables	*N*	Percentage (%)
Area		
Central area	801	17.5
Other	3,777	82.5
Gender		
Male	1,560	34.1
Female	3,018	65.9
Age		
≤ 45	2,336	51
45–60	1,341	29.3
≥60	901	19.7
With chronic diseases		
Yes	1,443	31.5
No	3,135	68.5
Duration of illness		
5–10 years	1,039	72
11–15 years	221	15.3
16–20 years	103	7.1
21–25 years	41	2.8
26–30 years	13	0.9
Over 30 years	26	1.8
Geographic accessibility		
Yes	2,402	52.5
No	2,176	47.5
Contract with family doctor		
Yes	4,176	91.2
No	402	8.8

The table presents the frequency distribution of the categorical characteristics of the study participants. Data are presented as n (percentage %). The analysis for “Duration of illness” was conducted exclusively among the subsample of participants who reported having chronic diseases (n = 1,443). Percentages may not sum to 100 due to rounding.

**Table 3:** Characteristics of participants (continuous variable).

Variables	M (IQR)	Possible ranges of the scores
Financial affordability	4	1–5
Realized accessibility	41 (10)	12–60
Availability	11 (4)	3–15
Acceptability	10 (3)	3–15
Accommodation	10 (3)	3–15
Accountability	11 (4)	3–15
Interactive interpersonal perceived quality	38 (11)	11–55
Perceived feasibility	14 (4)	4–20
Perceived desirability	13 (4)	4–20
Perceived continuity	11 (3)	3–15
Family doctor first-contact behavior	8 (3)	2–10

The table summarizes the descriptive statistics for the key continuous variables analyzed in the study. Given the skewed distribution of the data, all scores are presented as Median (Interquartile Range). The “Possible ranges of the scores” column indicates the theoretical minimum and maximum value for each measurement scale.

### 4.2 Psychometric properties of the scales

The psychometric assessment indicated that the items were suitable for factor analysis (KMO = 0.933; Bartlett's test of sphericity: χ^2^ = 77,566.1, df = 276, *p* < 0.001). The first four eigenvalues exceeded 1.0, indicating four dominant empirical factors. The rotated factor loading pattern nevertheless supported the overall multidimensional structure of the scale, although some theoretically distinct dimensions were empirically clustered. Inter-dimension correlations were generally consistent with the theoretical relationships among the constructs. Internal consistency was acceptable to excellent across the multi-item theoretical dimensions, with Cronbach's alpha ranging from 0.762 to 0.917; the overall scale alpha was 0.924. Full dimension-level reliability results, item-total statistics, rotated factor loadings, inter-dimension correlations, and eigenvalues are provided in the Appendix.

### 4.3 Multiple linear regression analysis

As shown in Table 4, after controlling for variables such as area gender, age, with chronic disease, and contract status, the overall explanatory power of the model was weak (Model 1 *R*^2^ = 0.002), and among the main control variables, only contract status had a significant impact on the FDFCB (*B* = 0.33, *p* = 0.003). Based on this, two core explanatory variables, geographical accessibility and financial affordability, were introduced (Model 2). The model fit did not significantly improve (Δ*R*^2^ = 0, *F*= 0.179, *p* = 0.836), indicating that these two policy-related accessibility indicators did not have a significant independent effect on patients' family doctor first-contact behavior after controlling for background factors. Hypothesis 1 was not supported, suggesting that residents' healthcare-seeking behavior may be more influenced by factors such as healthcare experience.

**Table 4:** Multiple linear regression results.

Variables	Model 1		Model 2	
	β	*t*	β	*t*
Area	−0.004	−0.048	−0.004	−0.043
Gender	0.002	0.025	0.001	0.011
Age	−0.062	−1.27	−0.061	−1.246
With chronic disease	0.011	0.042	0.013	0.052
Contract with family doctor	0.33	3.016	0.331	3.025^***^
Geographic Accessibility			−0.032	−0.515
Financial Affordability			0	0

Model 1: R^2^ = 0.002; F = 1.798, P = 0.095 Model 2: R^2^ = 0.002; F = 0.179, P = 0.836

The table presents the results of a hierarchical multiple linear regression analysis. Model 1 includes demographic, health, and contract status variables. Model 2 adds potential accessibility variables (Geographic Accessibility and Financial Affordability) to Model 1. The dependent variable is FDFCB. ^*^p < 0.05, ^**^p < 0.01, ^***^p < 0.001.

### 4.4 Latent profile analysis

To identify distinct subgroups of patients based on their IIPQ, we conducted a latent profile analysis, fitting models with one to seven profiles. Model fit indices are presented in Table 5. While information criteria (AIC, BIC, aBIC) continued to decrease with more profiles, and likelihood ratio tests (BLRT, LMR-LRT) remained significant for models with more than three profiles, the 3-profile model was selected as the optimal solution. This decision was primarily based on two key indicators: first, the entropy value reached its maximum (Entropy = 0.947) in the 3-profile model, indicating the highest classification accuracy; second, this model provided the most theoretically meaningful and interpretable latent profiles.

**Table 5:** Results of model fit, the accuracy of model classification and fit differences.

Model	AIC	BIC	aBIC	Entropy	LMR	BLRT	Class proportions
1	71,685.185	71,723.759	71,704.693				
2	65,670.168	65,734.458	65,702.682	0.990	0.0000	0.0000	0.90716/0.09284
3	59,768.149	59,858.156	59,813.669	0.947	0.0000	0.0000	0.08825/0.74356/0.16820
4	58,401.043	58,516.766	58,459.569	0.878	0.0000	0.0000	0.24880/0.08606/0.50612/0.15902
5	57,043.294	57,184.732	57,114.825	0.921	0.0000	0.0000	0.08912/0.01704/0.55505/0.19419/0.14460
6	56,180.979	56,348.134	56,265.516	0.878	0.0001	0.0000	0.01704/0.39450/0.08716/0.21822/0.14460/0.13849
7	55,315.993	55,508.864	55,413.535	0.908	0.0014	0.0000	0.08672/0.18567/0.42093/0.01704/0.13062/0.01616/0.14286

The table presents the model fit statistics used to determine the optimal number of latent profiles. Lower values for AIC, BIC, and aBIC indicate a better relative model fit. Entropy measures the classification accuracy, with values closer to 1 indicating clearer profile separation. The LMR and BLRT are likelihood ratio tests comparing the k-profile model with the k-1 profile model; a significant p-value (p <0.05) suggests that the k-profile model is a significant improvement. The 3-profile solution was selected as the optimal model based on a comprehensive evaluation of these indices, particularly its high entropy and theoretical interpretability.

**Figure 4:**
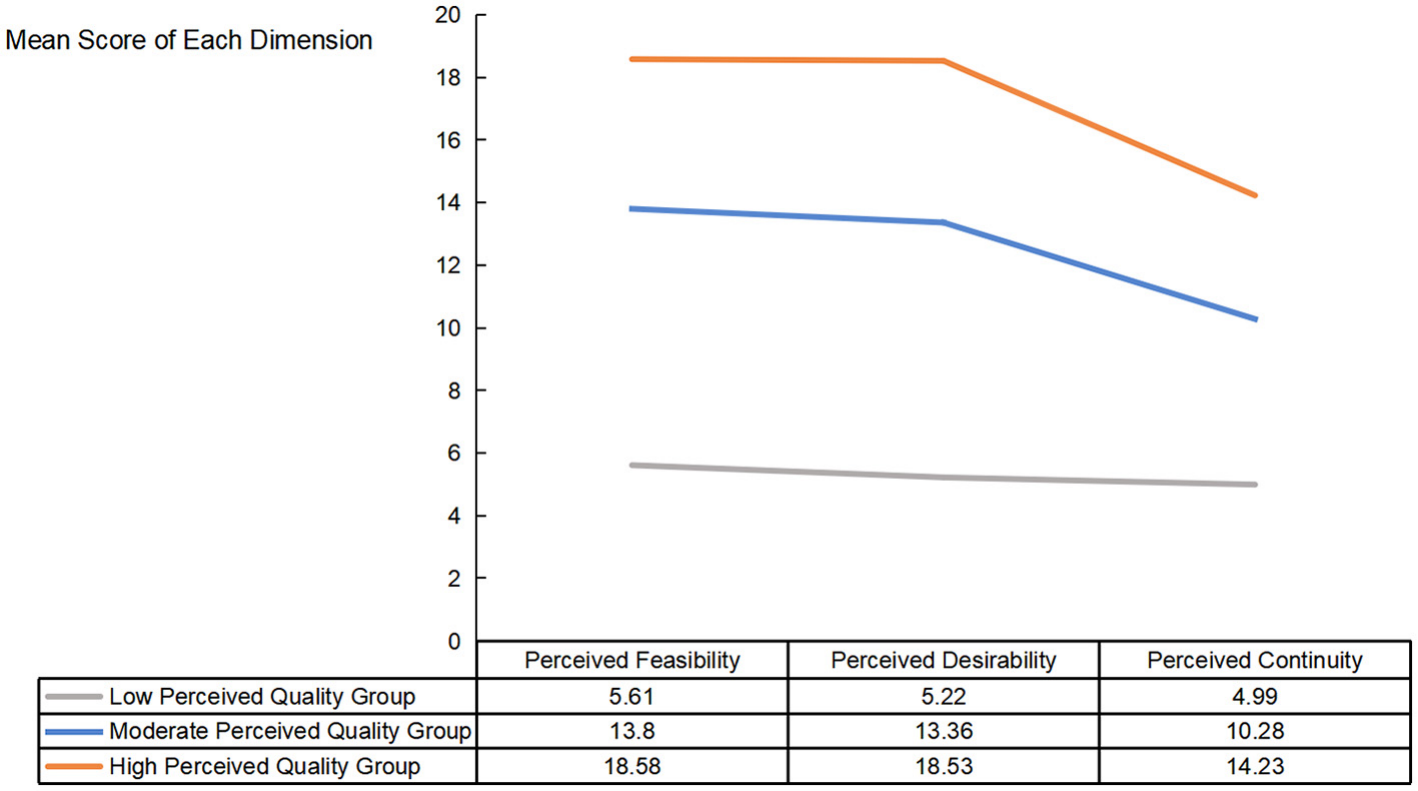
Mean score of the 3-latent-profile solution for Interactive Interpersonal Perceived Quality. The y-axis represents the mean score for each dimension, and the x-axis represents the three sub-dimensions of IIPQ. The lines depict the distinct patterns of mean scores for the three latent profiles identified by the Latent Profile Analysis: the Low Perceived Quality Group (gray line), the Moderate Perceived Quality Group (blue line), and the High Perceived Quality Group (orange line). The table below the plot provides the exact mean scores for each group on each dimension.

Figure 4 shows the average scores across three dimensions of perceived quality across three latent categories. We observe that the average score for each dimension follows the relationship: latent profile 3 >latent profile 2 >latent profile 1. We assigned the high perceived quality group, moderate perceived quality group, and low perceived quality group to latent categories 3, 2, and 1, respectively. The moderate perceived quality group constitutes the majority, accounting for approximately 74.4%, while the high perceived quality group and low perceived quality group account for approximately 8.8% and 16.8%, respectively.

### 4.5 Univariate analysis and binary logistic regression

**Figure 5:**
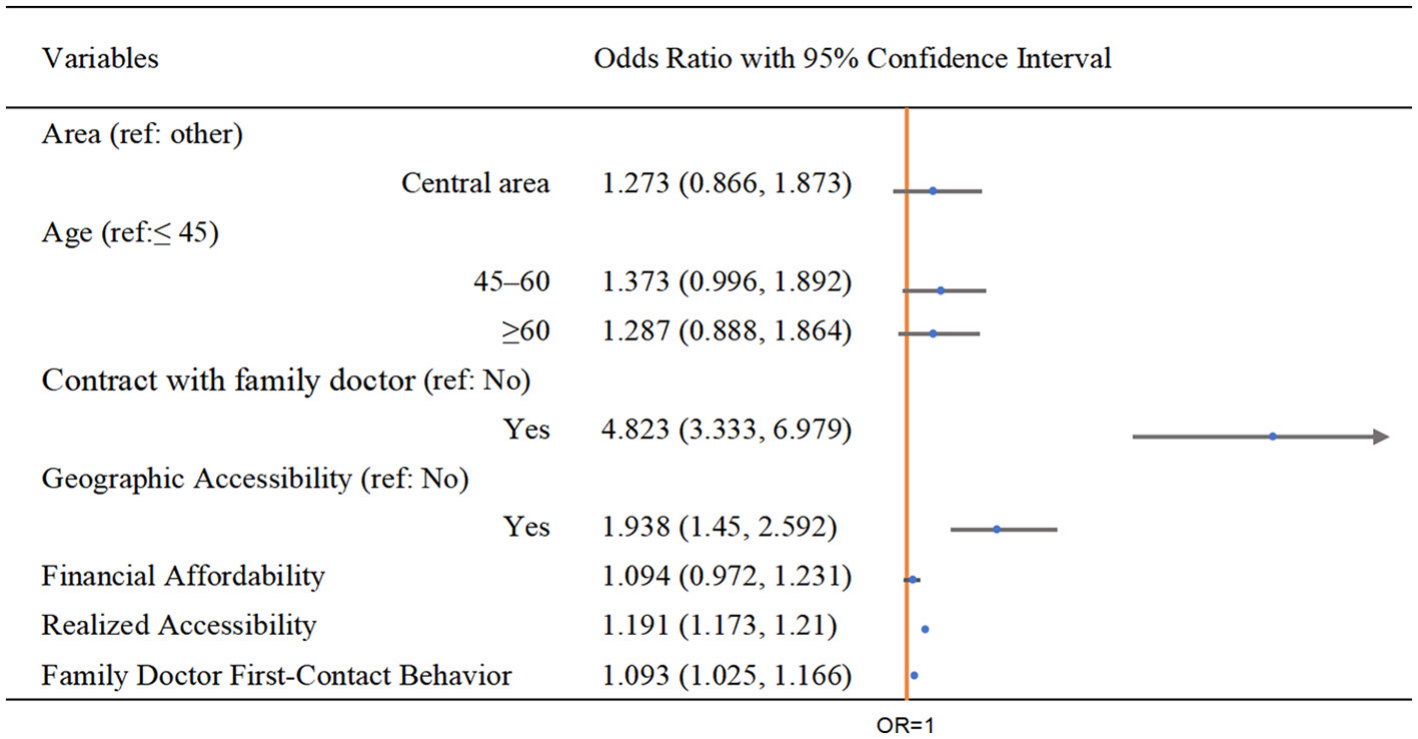
Forest plot of odds ratios from the logistic regression model predicting the likelihood of Interactive Interpersonal Perceived Quality. The plot displays the odds ratio (OR, blue dot) and its corresponding 95% confidence interval (CI, horizontal line) for each predictor variable. The vertical orange line represents an odds ratio of 1, indicating no effect. An odds ratio is considered statistically significant if its 95% confidence interval does not cross the line of no effect (OR = 1). Reference categories for categorical variables are indicated in parentheses.

Table 6 lists the results of the chi-square test. The analysis found significant differences among the three latent profile groups in the categorical variables of region, age, Geographic Accessibility, and contract with family doctor. In the continuous variables in Table 7, financial affordability, the total score and the four dimensions of realized accessibility, and the total score of patients' family doctor first-contact behavior showed significant differences across the three latent profile groups of perceived quality, indicating that these factors may lead to different perceived quality of family doctor contract services among residents, supporting Hypothesis 4. All variables significant in the univariate analysis were included in a binary logistic regression analysis, with results shown in Figure 5. Compared with residents who did not contract with family doctor services, those who did were more likely to belong to higher perceived quality groups, with odds ratios (OR) and 95% confidence intervals (CI) of 4.823 (3.333, 6.979), indicating that contract with family doctor services may be a protective factor for perceived quality. Residents who find it more convenient to access the nearest community health service station (Geographic Accessibility) (OR = 1.938, 95% CI = 1.45–2.592) are also more likely to belong to the higher perceived quality group. High realized accessibility and a stronger perceived orientation toward family doctors as first-contact providers were also associated with higher IIPQ, with OR and 95% CI of 1.191 (1.173, 1.21) and 1.093 (1.025, 1.166), respectively.

**Table 6:** Comparisons of latent profiles of general demographic data of residents (continuous variable).

Variables	Latent profile [*N* (%)]				
	Profile 1	Profile 2	Profile 3	Chi-square value	*p*
Area				19.516	<0.001
Central area	47 (13.9%)	719 (18.9%)	247 (13%)		
Other	357 (86.1%)	2,685 (81.1%)	523 (87%)		
Gender				2.359	0.307
Male	128 (31.7%)	1,181 (34.7%)	251 (32.6%)		
Female	276 (68.3%)	2,223 (65.3%)	519 (67.4%)		
Age				9.84	0.043
≤ 45	201 (49.8%)	1,743 (51.2%)	392 (50.9%)		
45–60	117 (29%)	1,023 (30.1%)	201 (26.1%)		
≥60	86 (21.3%)	638 (18.7%)	177 (23%)		
With chronic diseases				4.03	0.133
Yes	122 (30.2%)	1,055 (31%)	266 (34.5%)		
No	282 (69.8%)	2,349 (69%)	504 (65.5%)		
Duration of illness				16.85	0.078
5–10 years	84 (68.9%)	774 (73.4%)	181 (68%)		
11–15 years	19 (15.6%)	150 (14.2%)	52 (19.5%)		
16–20 years	13 (10.7%)	76 (7.2%)	14 (5.3%)		
21–25 years	6 (4.9%)	26 (2.5%)	9 (3.4%)		
26–30 years	0 (0%)	11 (1%)	2 (0.8%)		
30 years or more	0 (0%)	18 (1.7%)	8 (3%)		
Geographic accessibility				236.983	<0.001
Yes	210 (52%)	1,595 (46.9%)	597 (77.5%)		
No	194 (48%)	1,809 (53.1%)	173 (22.5%)		
Contract with family doctor				188.373	<0.001
Yes	341 (84.4%)	3,218 (94.5%)	617 (80.1%)		
No	63 (15.6%)	186 (5.5%)	153 (19.9%)		

The table displays the distribution of categorical demographic, health, and service-related characteristics across the three latent profiles identified by Latent Profile Analysis. The Pearson Chi-square test was used to examine the statistical significance of the differences in proportions across the three groups. Data are presented as n (%).

**Table 7:** Comparisons of latent profiles of general demographic data of residents (continuous variable).

Variables	Latent profile [M (IQR)]					
	Profile 1	Profile 2	Profile 3	*H*-value	*Post hoc* comparison	*p*
Financial affordability	4 (1)	4 (1)	4 (2)	17.2	1–2–3	<0.0001
Realized accessibility	12 (24)	41 (8)	45.5 (9)	774.714	1–2–3	<0.0001
Availability	3 (6)	11 (4)	12 (2)	507.313	1–2–3	<0.0001
Acceptability	3 (6)	10 (3)	12 (2)	630.464	1–2–3	<0.0001
Accommodation	3 (7)	10 (3)	12 (2)	508.501	1–2–3	<0.0001
Accountability	3 (6)	11 (4)	12 (2)	572.674	1–2–3	<0.0001
Family doctor first-contact behavior	5 (4)	8 (2)	9 (2)	334.037	1–2–3	<0.0001

The table displays the comparison of continuous study variables across the three latent profiles identified by Latent Profile Analysis. Due to the non-normal distribution of the data, the Kruskal–Wallis H-test was used for group comparisons. All variable scores are presented as Median (Interquartile Range). Post hoc pairwise comparisons revealed significant differences (p <0.05) between all three profiles for all listed variables.

### 4.6 The moderating effect of residents' perceived quality with different latent profiles

**Figure 6:**
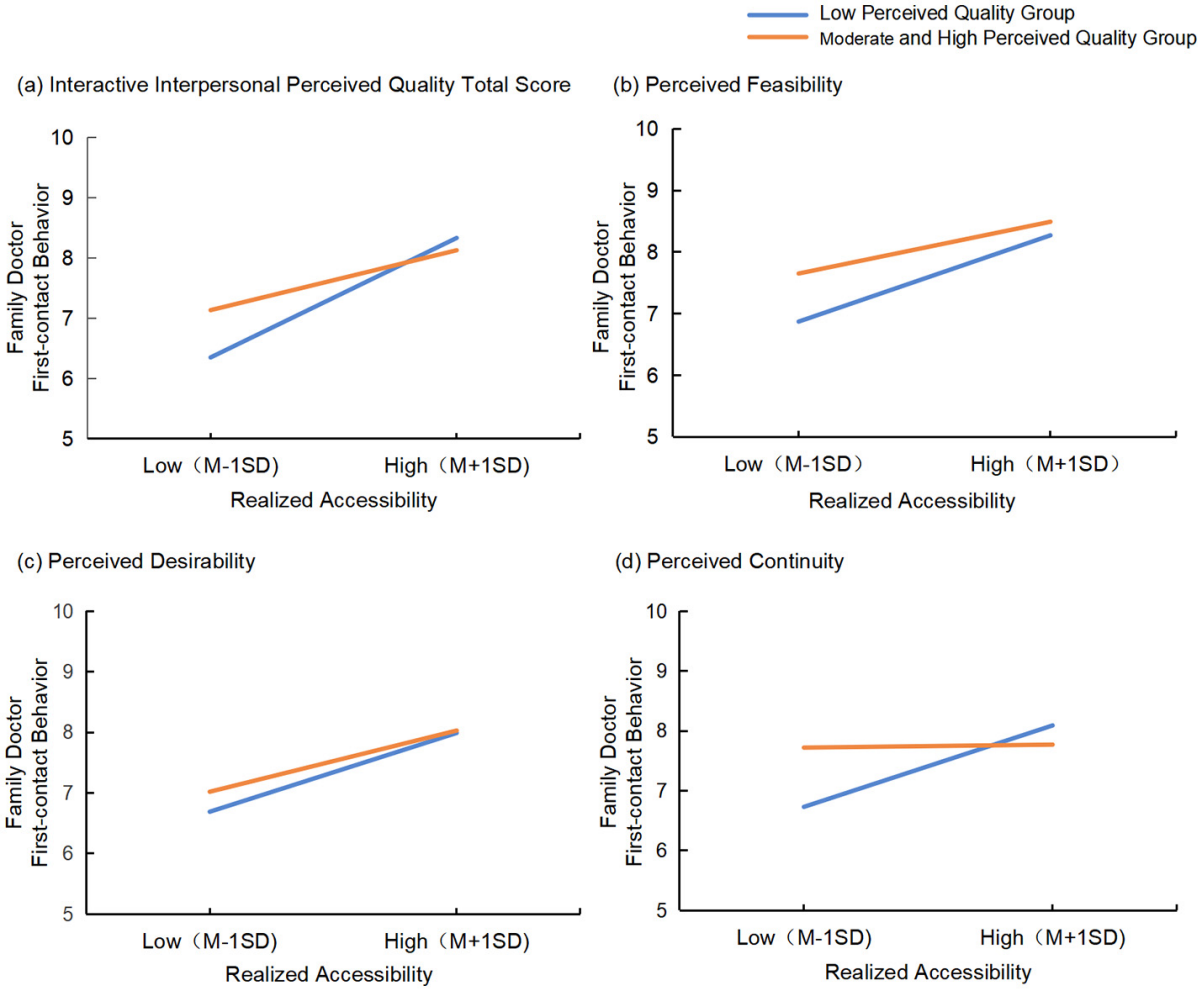
The difference effect from realized accessibility to family doctor first-contact behavior in different latent profile groups. The plots depict the predicted family doctor first-contact behavior at low (−1 SD) and high (+1 SD) levels of realized accessibility for two distinct groups: the Low Perceived Quality Group (blue line) and the Moderate and High Perceived Quality Group (orange line). **(a)** shows the moderating effect of the total score of Interactive Interpersonal Perceived Quality (IIPQ). **(b–d)** illustrate the specific moderating effects of the IIPQ sub-dimensions: perceived feasibility, perceived desirability, and perceived continuity, respectively.

To examine the moderating role of IIPQ in the relationship between realized accessibility and patients' FDFCB, this study adopted a stratified regression analysis method and included overall perception quality and its three sub-dimensions into the model for verification. The results of the stratified regression indicate that after adding the interaction term between the independent variables and overall perceived quality, the *R*-squared value increased from 0.139 to 0.146, indicating that the addition of the interaction term significantly enhanced the model's explanatory power (*F* = 35.178, *p* < 0.001), suggesting that the path from realized accessibility to patients' family doctor first-contact behavior is moderated by IIPQ. Hypotheses 2 and 3 are supported. As shown in Figure 6, in the higher perceived quality group, patients' family doctor first-contact behavior scores were higher, but the positive predictive effect of realized accessibility on family doctor first-contact behavior was relatively weaker compared to the lower perceived quality group, exhibiting a “ceiling effect.” Further dividing the IIPQ into three dimensions, the moderating effect persists but manifests differently. The moderating effects of perceived feasibility and perceived desirability are similar, and both positively moderate the relationship between realized accessibility and family doctor first-contact behavior, with the predictive effect of realized accessibility on family doctor first-contact behavior strengthened in the low perceived group. The moderating pattern of sustained perception differs: in the medium and high perceived quality group, the predictive effect of realized accessibility on primary healthcare-seeking is not significant, while it still has some influence in the low perceived quality group, suggesting that it weakens the role of external service convenience in stabilizing service relationships. Hypothesis 5 is supported.

## 5 Discussion

This study analyzed the complex mechanisms influencing the effectiveness of China's family doctor contract services. Using latent profile analysis, we revealed the heterogeneity within patient groups and their influencing factors, and further tested the moderating role of interactive interpersonal quality perceptions on patients' family doctor first-contact behavior. The results revealed the differences and mechanism logic in residents' healthcare-seeking behavior from multiple dimensions, providing important policy implications. Our conclusions are as follows:

Structural indicators are no longer important factors influencing residents' willingness to seek family doctor. Contrary to expectations, geographical accessibility and economic affordability did not have a significant impact on FDFCB. Traditional indicators of potential accessibility, which have been widely included in policy interventions, are no longer core factors determining residents' willingness to seek family doctor. Historically, policy design has primarily focused on enhancing the spatial equity of medical resources, emphasizing spatial coverage goals such as the “15-min healthcare circle,” with geographical accessibility viewed as a fundamental guarantee of service equity. However, research indicates that the idea of achieving equality and promoting healthcare utilization solely through potential accessibility based on distance remains overly narrow. Additionally, in recent years, Tianjin has implemented a family doctor payment system based on per capita fees, effectively reducing the economic burden on residents when signing up for the service, thereby alleviating the constraints on initial healthcare-seeking behavior imposed by economic affordability at the institutional level. Accessibility is a multidimensional and dynamic concept in the process of healthcare service utilization and cannot be simply equated with spatial barriers or economic costs. Accessibility should be defined as the opportunity to access and obtain healthcare services, service providers, or institutions when deemed necessary (44, 45). Therefore, research should go beyond traditional geographical and cost indicators to gain a deeper understanding of how residents perceive, evaluate, and prioritize healthcare services, focusing on understanding the role of service quality, doctor-patient relationships, and user experience from the residents' subjective perspective.

There is significant heterogeneity in the interactive interpersonal perception quality among resident groups. Previous studies have found that residents' perceived quality is generally at a moderate level (46), and have described residents' perceived quality levels as overall levels. This study identified three groups of perceived quality through latent feature analysis: high perceived quality group (8.8%), medium perceived quality group (74.4%), and low perceived quality group (16.8%). It can be seen that the medium-perceived quality group dominates in terms of numbers, but this overall moderate evaluation may precisely mask the key characteristics of marginal groups, thereby affecting the true impact on perceptions of service effectiveness. This result suggests that although the family doctor contract system has achieved widespread coverage in form, there are significant differences between residents' acceptance, trust, and experience of the services, reflecting a certain gap between institutional supply and residents' perceptions. Such heterogeneous phenomena pose challenges to traditional policy performance evaluation methods, especially since most current evaluations use the contract rate as the core indicator, lacking in-depth analysis of service quality perceptions and usage experiences, and thus unable to accurately measure the actual effectiveness of the system.

Traditionally perceived objective resource allocation is not the primary driver of differences in service quality perceptions. To uncover the mechanisms underlying group differences, this study further employed univariate analysis and binary logistic regression to examine the association between resident characteristics and their perceived quality group affiliation. Univariate analysis results showed that region, age, geographical accessibility, contract with family doctor, financial affordability, realized accessibility total score, all dimensions of realized accessibility, and the total score of FDFCB were all significantly associated. When these factors were included in the binary logistic regression, only having a contract with family doctor (Yes, reference: No), geographical accessibility, financial affordability, realized accessibility total score, and patients' FDFCB were significant, indicating that these factors may be associated with perceived quality. Notably, despite long-standing imbalances in the objective allocation of medical resources in suburban areas, our study found no significant differences in the IIPQ of family doctor contract services between central area residents and residents of other regions, which differs from previous research (11). One possible explanation is that perceived quality is not only influenced by actual service performance but also by patient expectations, which may vary across different populations (17, 47). Residents outside central areas may have historically had lower expectations for services, so even when actual service performance is average, their perceived quality may be relatively high; in contrast, central area residents often have higher expectations and are more critical of service experiences. This supports the expectation-confirmation theory of service quality evaluation (48, 49). This finding challenges the traditional assumption of cross-regional perceived inequality. From a policy perspective, this suggests that efforts to further improve the perceived quality of family doctor contract services should consider internal heterogeneity within groups rather than focusing solely on regional differences. Additionally, overall perceived quality may mask differences in its underlying dimensions. Although residents may have similar overall scores, future studies should investigate whether residents differ in their perceptions of quality across different sub-dimensions. In summary, the results of the binary logistic regression can assist policymakers in identifying residents with poor perceived quality.

IIPQ significantly moderates the relationship between realized accessibility and FDFCB. In the moderating effects of overall perceived quality and its three sub-dimensions, the intercepts are smaller for groups with lower perceived quality and larger for groups with higher perceived quality, indicating that residents in the medium-to-high perceived quality groups maintain a stronger perceived first-contact orientation toward family doctors even under low realized accessibility conditions. The slope is steeper in the low perceived quality group, indicating that improvements in realized accessibility have a more significant impact on family doctor first-contact behavior; while the slope is relatively flat in the medium-high perceived quality group, suggesting that residents' family doctor first-contact behavior is less dependent on realized accessibility. This phenomenon is more pronounced in the continuity dimension. Although previous studies have generally recognized that realized accessibility significantly influences residents' FDFCB (50), the mechanism through which perceived quality moderates this relationship has been less explored. This study further validated the moderating role of residents' IIPQ, particularly sustained perception, between realized accessibility and family doctor first-contact behavior through quantitative empirical analysis, revealing from the demand side how perceptual factors indirectly influence residents' healthcare preference. This mechanism provides a new perspective on the phenomenon of “sign but not consult,” suggesting that even if residents complete the signing process, they may still struggle to establish a behavioral tendency to use family doctors as their primary care entry point if their subjective perceptions of service continuity are insufficient. The study found that whether residents have signed up for family doctor services is an important factor influencing their IIPQ. Previous studies have confirmed that signing up for such services can, to some extent, increase residents' likelihood of choosing family doctors (51, 52), but have not explored the underlying pathways. This study points out that the key to this impact lies in whether the signing of a contract can bring about a continuous service experience. Residents who have not signed a contract often find it difficult to form long-term trust due to the lack of stable and continuous healthcare relationships (53, 54), and the lack of such service continuity also weakens residents' recognition of the comprehensive service capabilities of family doctors. This may be because unsigned residents often lack stable, continuous service experiences, and residents have not established long-term relationships with family doctors. Family doctors, lacking knowledge of residents' previous personal conditions, are unable to better understand their health issues and individual needs. In the family doctor service system, continuity is an important dimension for measuring service quality, indicating whether doctors continuously monitor patients' health conditions and provide continuous care support. After signing up, residents are usually included in the family doctor's active management system, which includes regular physical examinations, medication follow-ups, health guidance, and psychological support. This long-term, high-frequency service arrangement helps deepen residents' sense of recognition and dependence on family doctors, forming a stable preference for seeking medical treatment, thereby increasing the actual utilization rate of family doctors as the first point of contact. Therefore, continuous perception is not only the result of the service experience but also the starting point for influencing residents' behavioral choices. Compared with the supply dimension, which focuses on the adequacy of medical service resources, the perceived quality of residents' needs better reflects the real connection between institutional design and residents' health behavior.

It is worth noting that in this study, more than 15% of residents still fall into the low perception quality group. Most of them have not yet established stable contract relationships and are unable to build positive expectations of service quality based on personal experience. For this group, realized accessibility is the primary driver of behavioral change. Therefore, policies should prioritize further improving service realized accessibility for this group to meet residents' needs for basic services. On this basis, as service contact frequency and quality improve, residents will gradually develop positive interactive interpersonal perceptions of the family doctor contract services, ultimately leading to more stable consult preferences. For groups with higher perceived quality, intervention efforts should focus on optimizing service experiences, (55) thereby enhancing service stickiness, extending service relationships, and increasing residents' sustained reliance on the family doctor contract services.

### 5.1 Limitations

This study has several limitations that should be considered. First, the survey data is entirely based on self-reports, which may introduce recall bias. Second, the cross-sectional study only provides a snapshot of perceived quality at a specific point in time. Longitudinal studies are needed to track whether residents' perceived quality changes following policy interventions. Third, although the sample size is large, the size of the identified subgroups is unbalanced, which may affect the analysis results. In summary, this study provides preliminary evidence for grouping residents based on perceived quality, but further research is needed to validate and expand these findings.

## 6 Conclusion

The findings of the perception-behavior conversion in this study provide new practical directions for policy interventions in FDCS. This study focuses on the interactive interpersonal perception quality formed in doctor-patient interactions, identifies heterogeneity in residents' perception quality through latent category analysis, and analyzes the moderating mechanisms of the three types of perception in influencing residents' FDFCB. The study found that residents exhibit three distinct types of perception quality, with the largest group having moderate perception quality, while smaller groups exhibit high and low perception quality. Residents who have contracted with family doctors and those who find it convenient to visit the nearest community health service station are more likely to belong to the higher perception quality group. The moderating effects further revealed significant differences in the pathways through which realized accessibility influenced FDFCB across different perceived quality groups. These findings suggest that when designing policies to promote the FDCS, policymakers should avoid a balanced supply model and instead adopt a tiered intervention and targeted incentive strategy based on the level of perceived quality.

## Data Availability

The raw data supporting the conclusions of this article will be made available by the authors, without undue reservation.

## References

[1] Shao S Wang M Jin G Zhao Y Lu X Du J. Analysis of health service utilization of migrants in Beijing using Anderson health service utilization model. BMC Health Serv Res. (2018) 18:462. doi: 10.1186/s12913-018-3271-y 29914464 PMC6006712

[2] Giusti M De Remigis S Greco S Profeta VF Bonaccorsi G Zanobini P . Integration of resources in social and healthcare services for ensuring the continuity of care to frail individuals aged 65 or over: an Italian experience. Front Public Health. (2025) 13:1562564. doi: 10.3389/fpubh.2025.1562564 40469610 PMC12135681

[3] Sousa L Käppler C Faleiros F Alburquerque G José H. Management of chronic health situations. Healthcare. (2026) 14:176. doi: 10.3390/healthcare14020176 41595312 PMC12840840

[4] Shi J Jin H Shi L Chen C Ge X Lu Y . The quality of primary care in community health centers: comparison among urban, suburban and rural users in Shanghai, China. BMC Fam Pract. (2020) 21:178. doi: 10.1186/s12875-020-01250-6 32854623 PMC7453522

[5] Li X Krumholz HM Yip W Cheng KK Maeseneer JD Meng Q . Quality of primary health care in China: challenges and recommendations. Lancet. (2020) 395:1802–12. doi: 10.1016/S0140-6736(20)30122-7 32505251 PMC7272159

[6] Wu Y Ye R Wang Q Sun C Meng S Sylvia S . Provider competence in hypertension management and challenges of the rural primary healthcare system in Sichuan province, China: a study based on standardized clinical vignettes. BMC Health Serv Res. (2022) 22:849. doi: 10.1186/s12913-022-08179-9 35778732 PMC9248120

[7] Yuan S Wang F Li X Jia M Tian M. Facilitators and barriers to implement the family doctor contracting services in China: findings from a qualitative study. BMJ Open. (2019) 9:e032444. doi: 10.1136/bmjopen-2019-032444 31597653 PMC6797329

[8] Liao R Liu Y Peng S Feng XL. Factors affecting health care users' first contact with primary health care facilities in north eastern China, 2008–2018. BMJ Glob Health. (2021) 6:e003907. doi: 10.1136/bmjgh-2020-003907 33597277 PMC7893657

[9] Xu J Mills A. Challenges for gatekeeping: a qualitative systems analysis of a pilot in rural China. Int J Equity Health. (2017) 16:106. doi: 10.1186/s12939-017-0593-z 28666445 PMC5493841

[10] Rao KD Peters DH Bandeen-Roche K. Towards patient-centered health services in India—a scale to measure patient perceptions of quality. Int J Qual Health Care. (2006) 18:414–21. doi: 10.1093/intqhc/mzl049 17012306

[11] Tang L. The influences of patient's trust in medical service and attitude towards health policy on patient's overall satisfaction with medical service and sub satisfaction in China. BMC Public Health. (2011) 11:472. doi: 10.1186/1471-2458-11-472 21676228 PMC3129314

[12] Zhao N Gu M Li J Zhang H Yang J. Factors influencing contracting of residents with family doctors in China: a national cross-sectional survey. BMC Health Serv Res. (2024) 24:213. doi: 10.1186/s12913-024-10606-y 38360648 PMC10870580

[13] Cheraghi-Sohi S Bower P Mead N McDonald R Whalley D Roland M. What are the key attributes of primary care for patients? Building a conceptual ‘map' of patient preferences. Health Expect. (2006) 9:275–84. doi: 10.1111/j.1369-7625.2006.00395.x 16911142 PMC5060357

[14] Goins RT Williams KA Carter MW Spencer SM Solovieva T. Perceived barriers to health care access among rural older adults: a qualitative study. J Rural Health. (2005) 21:206–13. doi: 10.1111/j.1748-0361.2005.tb00084.x 16092293

[15] Edelman MA Menz BL. Selected comparisons and implications of a national rural and urban survey on health care access, demographics, and policy issues. J Rural Health. (1996) 12:197–205. doi: 10.1111/j.1748-0361.1996.tb00794.x 10162851

[16] Andersen RM McCutcheon A Aday LA Chiu GY Bell R. Exploring dimensions of access to medical care. Health Serv Res. (1983) 18:49–74. 6841113 PMC1068709

[17] Weinhold I Gurtner S. Rural-urban differences in determinants of patient satisfaction with primary care. Soc Sci Med. (2018) 212:76–85. doi: 10.1016/j.socscimed.2018.06.019 30025382

[18] Huang Y Meyer P Jin L. Spatial access to health care and elderly ambulatory care sensitive hospitalizations. Public Health. (2019) 169:76–83. doi: 10.1016/j.puhe.2019.01.005 30825835

[19] Zhao L Huiqin Y. Impact of healthcare accessibility on the quality of life of older adults in china and its age differences. SAGE Open. (2025) 15:3. doi: 10.1177/21582440251356051

[20] Tang C Luo Z Fang P Zhang F. Do patients choose community health services (CHS) for first treatment in China? Results from a Community Health Survey in Urban Areas. J Community Health. (2013) 38:864–72. doi: 10.1007/s10900-013-9691-z 23636415

[21] Yip W Hsiao W. China's health care reform: a tentative assessment. China Econ Rev. (2009) 20:613–9. doi: 10.1016/j.chieco.2009.08.003

[22] Sun X Meng H Ye Z Conner KO Duan Z Liu D. Factors associated with the choice of primary care facilities for initial treatment among rural and urban residents in Southwestern China. PLoS ONE. (2019) 14:e0211984. doi: 10.1371/journal.pone.0211984 30730967 PMC6366770

[23] Penchansky R Thomas JW. The Concept of access: definition and relationship to consumer satisfaction. Med Care. (1981) 19:127. doi: 10.1097/00005650-198102000-00001 7206846

[24] National Health Commission of the People's Republic of China. Opinions on Implementation of Family Doctor Contracted Services Policy 2019. Beijing: National Health Commission of the People's Republic of China. (2019).

[25] State Council of the People's Republic of China. Guidance on Regulating the Implementation of Family Doctor Contracted Services. Beijing: State Council of the People's Republic of China. Available online at: https://www.gov.cn/zhengce/zhengceku/2018-12/31/content_5435461.htm (Accessed May 29, 2025).

[26] Tilahun D Hanlon C Araya M Davey B Hoekstra RA Fekadu A. Training needs and perspectives of community health workers in relation to integrating child mental health care into primary health care in a rural setting in sub-Saharan Africa: a mixed methods study. Int J Ment Health Syst. (2017) 11:15. doi: 10.1186/s13033-017-0121-y 28168004 PMC5286789

[27] Loyola-Sanchez A Richardson J Wilkins S Lavis JN Wilson MG Alvarez-Nemegyei J . Barriers to accessing the culturally sensitive healthcare that could decrease the disabling effects of arthritis in a rural Mayan community: a qualitative inquiry. Clin Rheumatol. (2016) 35:1287–98. doi: 10.1007/s10067-015-3061-4 26334916

[28] Levesque J-F Harris MF Russell G. Patient-centred access to health care: conceptualising access at the interface of health systems and populations. Int J Equity Health. (2013) 12:18. doi: 10.1186/1475-9276-12-18 23496984 PMC3610159

[29] Donabedian A. The quality of care: how can it be assessed? JAMA. (1988) 260:1743–8. doi: 10.1001/jama.1988.03410120089033 3045356

[30] Altman I Taylor DA. Social Penetration: The Development of Interpersonal Relationships. Oxford, England: Holt, Rinehart & Winston. (1973).

[31] Valderas JM Porter I Martin-Delgado J Rijken M de Jong J Groene O . Development of the patient-reported indicator surveys (PaRIS) conceptual framework to monitor and improve the performance of primary care for people living with chronic conditions. BMJ Qual Saf. (2025) 34:529–36. doi: 10.1136/bmjqs-2024-017301 39174334

[32] Herzberg F. One more time: how do you motivate employees? Harvard Bus Rev. (1968) 46:53–62. 12545925

[33] Proctor E Silmere H Raghavan R Hovmand P Aarons G Bunger A . Outcomes for implementation research: conceptual distinctions, measurement challenges, and research agenda. Adm Policy Ment Health Ment Health Serv Res. (2011) 38:65–76. doi: 10.1007/s10488-010-0319-7 20957426 PMC3068522

[34] Hall MA Camacho F Dugan E Balkrishnan R. Trust in the medical profession: conceptual and measurement issues. Health Serv Res. (2002) 37:1419–39. doi: 10.1111/1475-6773.01070 12479504 PMC1464022

[35] Hill KM Twiddy M Hewison J House AO. Measuring patient-perceived continuity of care for patients with long-term conditions in primary care. BMC Fam Pract. (2014) 15:191. doi: 10.1186/s12875-014-0191-8 25477059 PMC4264317

[36] Xu J Powell-Jackson T Mills A. Effectiveness of primary care gatekeeping: difference-in-differences evaluation of a pilot scheme in China. BMJ Glob Health. (2020) 5:2792. doi: 10.1136/bmjgh-2020-002792 32792410 PMC7430328

[37] Li C Chen Z Khan MM. Bypassing primary care facilities: health-seeking behavior of middle age and older adults in China. BMC Health Serv Res. (2021) 21:895. doi: 10.1186/s12913-021-06908-0 34461884 PMC8406824

[38] Haggerty JL Roberge D Lévesque J-F Gauthier J Loignon C. An exploration of rural–urban differences in healthcare-seeking trajectories: implications for measures of accessibility. Health Place. (2014) 28:92–8. doi: 10.1016/j.healthplace.2014.03.005 24793139

[39] Li Y-N Nong D Wei B Feng Q-M Luo H. The impact of predisposing, enabling, and need factors in utilization of health services among rural residents in Guangxi, China. BMC Health Serv Res. (2016) 16:592. doi: 10.1186/s12913-016-1825-4 27760531 PMC5070132

[40] Schäfer WLA Boerma WGW Schellevis FG Groenewegen PP GP. Practices as a one-stop shop: how do patients perceive the quality of care? A cross-sectional study in thirty-four countries. Health Serv Res. (2018) 53:2047–63. doi: 10.1111/1475-6773.12754 29285763 PMC6051984

[41] He Z Yang S Liu Y Yin L Li Z Weng Q. The effect of course characteristics and self-efficacy on practical training course satisfaction: moderating effect of the perceived usefulness of wisdom teaching. Sustainability. (2022) 14:15660. doi: 10.3390/su142315660

[42] Riva F Magrizos S Rubel MRB Rizomyliotis I. Green consumerism, green perceived value, and restaurant revisit intention: millennials' sustainable consumption with moderating effect of green perceived quality. Bus Strategy Environ. (2022) 31:2807–19. doi: 10.1002/bse.3048

[43] Hu R Liao Y Du Z Hao Y Liang H Shi L. Types of health care facilities and the quality of primary care: a study of characteristics and experiences of Chinese patients in Guangdong Province, China. BMC Health Serv Res. (2016) 16:335. doi: 10.1186/s12913-016-1604-2 27484465 PMC4969734

[44] Daniels N. Equity of access to health care: some conceptual and ethical issues. Milbank Mem Fund Q Health Soc. (1982) 60:51–81. doi: 10.2307/3349700 7038534

[45] Whitehead M. The concepts and principles of equity and health. Health Promot Int. (1991) 6:217–28. doi: 10.1093/heapro/6.3.217 1644507

[46] Liu S Meng W Yu Q Peng H Jiang X Li Z . Evaluation and countermeasures of contracted services of Chinese family doctors from demanders' point of view—a case study of a city. BMC Health Serv Res. (2022) 22:1534. doi: 10.1186/s12913-022-08891-6 36527029 PMC9758818

[47] Sitzia J Wood N. Patient satisfaction: a review of issues and concepts. Soc Sci Med. (1997) 45:1829–43. doi: 10.1016/S0277-9536(97)00128-7 9447632

[48] Parasuraman A Zeithaml VA Berry LL. Servqual: a multiple-item scale for measuring consumer perceptions of service quality. J Retail. (1988) 64:12–40

[49] Zeithaml VA Berry LL Parasuraman A. The nature and determinants of customer expectations of service. J Acad Mark Sci. (1993) 21:1–12. doi: 10.1177/0092070393211001

[50] Zheng Q Shi L Pang T Leung W. Utilization of community health care centers and family doctor contracts services among community residents: a community-based analysis in shenzhen, China. BMC Fam Pract. (2021) 22:100. doi: 10.1186/s12875-021-01444-6 34030650 PMC8146992

[51] Fu P Wang Y Zhao D Yang S Zhou C. Does contracting family doctor promote primary healthcare utilization among older adults? -evidence from a difference-in-differences analysis. BMC Geriatr. (2024) 24:749. doi: 10.1186/s12877-024-05336-z 39256643 PMC11385809

[52] Wang S Feng C Junfang X. The impact of family doctor contract services on the utilization of and satisfaction with primary health care among Chinese residents: a cross-sectional study. J Fam Med Primary Care. (2024) 13:1887–93. doi: 10.4103/jfmpc.jfmpc_1724_23 38948628 PMC11213444

[53] Kon AA. The shared decision-making continuum. JAMA. (2010) 304:903–4. doi: 10.1001/jama.2010.1208 20736477

[54] Ekman I Swedberg K Taft C Lindseth A Norberg A Brink E . Person-centered care — ready for prime time. Eur J Cardiovasc Nurs. (2011) 10:248–51. doi: 10.1016/j.ejcnurse.2011.06.008 21764386

[55] Li L Zhong C Mei J Liang Y Li L Kuang L. Effect of family practice contract services on the quality of primary care in Guangzhou, China: a cross-sectional study using PCAT-AE. BMJ Open. (2018) 8:e021317. doi: 10.1136/bmjopen-2017-021317 30420344 PMC6252701

[56] Hoseini-Esfidarjani S-S Negarandeh R Delavar F Janani L. Psychometric evaluation of the perceived access to health care questionnaire. BMC Health Serv Res. (2021) 21:638. doi: 10.1186/s12913-021-06655-2 34215250 PMC8254360

[57] Yang H Shi L Lebrun LA Zhou X Liu J Wang H. Development of the Chinese primary care assessment tool: data quality and measurement properties. Int J Qual Health Care. (2013) 25:92–105. doi: 10.1093/intqhc/mzs072 23175535

[58] Weiner BJ Lewis CC Stanick C Powell BJ Dorsey CN Clary AS . Psychometric assessment of three newly developed implementation outcome measures. Implement Sci. (2017) 12:108. doi: 10.1186/s13012-017-0635-3 28851459 PMC5576104

[59] Chao J. Continuity of care: incorporating patient perceptions. Fam Med. (1988) 20:333–7. 3266158

