# Supplementary material for: Why do they sign but not consult? A study on the effectiveness of China's family doctor contract services from the perspective of patient-doctor interaction
Source: Front Med (Lausanne). 2026 Aug 4;13:1848658. doi: 10.3389/fmed.2026.1848658 (PMC13481298; doi:10.3389/fmed.2026.1848658)
Supplement: Supplementary file 1 [file Data_Sheet_1.pdf]

Supplementary Material

1 Questionnaire

Questionnaire on Family Doctor Contract Services and Health Service Experience

This questionnaire aims to understand residents' experiences with family doctor contract services and their healthcare-seeking preferences. There are no right or wrong answers. Please choose the option that best reflects your actual situation and personal experience. Your responses will be used for research purposes only and will remain confidential.

Part A. Basic Information

1. Area of residence

□ Heping District

□ Hedong District

□ Hexi District

□ Nankai District

□ Hebei District

□ Hongqiao District

□ Dongli District

□ Xiqing District

□ Jinnan District

□ Beichen District

□ Wuqing District

□ Baodi District

□ Ninghe District

□ Jinghai District

□ Jizhou District

□ Binhai New Area

2. Gender

□ Male

□ Female

3. Age

_____ years old

4. Do you have any chronic disease?

□ Yes

□ No

5. If yes, how long have you had the chronic disease?

□ 5-10 years

□ 11-15 years

□ 16-20 years

□ 21-25 years

□ 26-30 years

□ More than 30 years

□ Not applicable

6. Have you contracted with a family doctor?

□ Yes

□ No

Part B. Potential Accessibility

7. Is it convenient for you to walk from your home to the nearest community health service centre (or a primary care institution providing family doctor services) (approximately within 15 minutes)?

□ Yes

□ No

Please indicate the extent to which you agree with each statement: 1 = Strongly disagree; 2 = Disagree; 3 = Neutral; 4 = Agree; 5 = Strongly agree.

8. Do you consider the current family doctor contract services to be affordable for you?

□ 1 □ 2 □ 3 □ 4 □ 5

Part C. Realized Accessibility

Please indicate the extent to which you agree with each statement: 1 = Strongly disagree; 2 = Disagree; 3 = Neutral; 4 = Agree; 5 = Strongly agree.

Questionnaire item	1	2	3	4	5
9. The services provided by family doctors generally meet my health needs.					
10. The facilities provided by family doctors are sufficient to meet my needs.					
11. There are sufficient numbers of family doctors available in the community to provide family doctor services.					
12. Family doctors are neatly dressed and professional.					
13. The clinic environment for family doctor services is comfortable, clean, and convenient.					
14. Family doctors can accurately record my information and maintain confidentiality.					
15. The process of receiving family doctor services is simple and efficient.					
16. Family doctors offer multiple communication methods (e.g., phone, online consultation, etc.).					
17. Family doctors flexibly arrange appointment times according to my specific needs.					
18. Family doctors proactively follow up on my health status.					
19. If I have further needs, family doctors will help me coordinate referrals.					
20. Family doctors can remember my medical history and treatment arrangements.					

Part D. Interactive Interpersonal Perceived Quality

Please indicate the extent to which you agree with each statement: 1 = Strongly disagree; 2 = Disagree; 3 = Neutral; 4 = Agree; 5 = Strongly agree.

Questionnaire item	1	2	3	4	5
21. I know how to contact family doctors.					
22. I believe that family doctor services are feasible under current conditions.					
23. I believe that family doctors can address my common minor health issues.					
24. Family doctor services are convenient for me to use.					
25. I believe that the advice provided by family doctors is trustworthy.					

26. I feel that family doctors are extremely thorough and attentive.					
27. The services provided by family doctors make me feel respected and understood.					
28. I am willing to contact family doctors because I believe they will provide me with a comprehensive treatment plan.					
29. Family doctors are very familiar with my situation and that of my family members.					
30. I am willing to maintain a long-term relationship with family doctors.					
31. I have established a stable communication mechanism with family doctors.					

Part E. Family Doctor First-Contact Behavior

Please indicate the extent to which you agree with each statement: 1 = Strongly disagree; 2 = Disagree; 3 = Neutral; 4 = Agree; 5 = Strongly agree.

**Questionnaire item**	**1**	**2**	**3**	**4**	**5**
32. I am able to proactively contact family doctors for medical consultation when needed.					
33. When experiencing non-emergency health problems, I first seek care from family doctors.					

2 中文版问卷

家庭医生签约服务与居民就医体验调查问卷

本问卷旨在了解居民对家庭医生签约服务的体验及就医选择偏好。所有问题没有对错之分。请您选择最能反映您实际情况和个人感受的选项。您的回答仅用于研究目的，并将予以保密。

第一部分 基本信息

1. 您的居住区域

□ 和平区

□ 河东区

□ 河西区

□ 南开区

□ 河北区

□ 红桥区

□ 东丽区

□ 西青区

□ 津南区

□ 北辰区

□ 武清区

□ 宝坻区

□ 宁河区

□ 静海区

□ 蓟州区

□ 滨海新区

2. 您的性别

□ 男

□ 女

3. 您的年龄

____ 岁

4. 您是否患有慢性病？

□ 是

□ 否

5. 如果患有慢性病，您的患病时长为：

□ 5-10 年

□ 11-15 年

□ 16-20 年

□ 21-25 年

□ 26-30 年

□ 30 年以上

□ 不适用

6. 您是否签约家庭医生？

□ 是

□ 否

第二部分 潜在可及性

7. 从您家步行到最近的社区卫生服务中心（或提供家庭医生服务的基层医疗卫生机构）是否方便（约 15 分钟以内）？

□ 是

□ 否

请根据您的实际感受选择同意程度：1 = 非常不同意；2 = 不同意；3 = 一般；4 = 同意；5 = 非常同意。

8. 您认为目前家庭医生签约服务的费用对您而言是可负担的吗？

□ 1　□ 2　□ 3　□ 4　□ 5

第三部分 实现可及性

请根据您的实际感受选择同意程度：1 = 非常不同意；2 = 不同意；3 = 一般；4 = 同意；5 = 非常同意。

**题项**	**1**	**2**	**3**	**4**	**5**
9. 家庭医生提供的服务总体上能够满足我的健康需求。					
10. 家庭医生提供的设施设备能够满足我的需求。					
11. 社区中有足够数量的家庭医生能够提供家庭医生服务。					
12. 家庭医生着装整洁，表现专业。					
13. 家庭医生的诊疗环境舒适、干净且便利。					
14. 家庭医生能够准确记录我的信息并保护隐私。					
15. 家庭医生的流程简单且高效。					
16. 家庭医生提供多种沟通方式（如电话、线上咨询等）。					
17. 家庭医生能够根据我的具体需求灵活安排预约时间。					
18. 家庭医生会主动随访我的健康状况。					
19. 如果我有进一步需求，家庭医生会帮助我协调转诊。					
20. 家庭医生能够记住我的病史和治疗安排。					

第四部分 互动性人际感知质量

请根据您的实际感受选择同意程度：1 = 非常不同意；2 = 不同意；3 = 一般；4 = 同意；5 = 非常同意。

**题项**	**1**	**2**	**3**	**4**	**5**
21. 我知道如何联系家庭医生。					
22. 家庭医生服务对我来说是可行的。					
23. 我认为家庭医生能够处理我常见的小病。					
24. 家庭医生服务对我来说使用起来很方便。					
25. 我认为家庭医生的建议值得信任。					
26. 我觉得家庭医生非常细致且认真。					
27. 家庭医生提供的服务让我感到被尊重和被理解。					
28. 我愿意联系家庭医生，因为我相信他们会为我提供全面的治疗方案。					
29. 家庭医生非常熟悉我及我家庭成员的情况。					

30. 我愿意与家庭医生保持长期关系。					
31. 我已经与家庭医生建立了稳定的沟通机制。					

第五部分 家庭医生首诊行为

请根据您的实际感受选择同意程度：1 = 非常不同意；2 = 不同意；3 = 一般；4 = 同意；5 = 非常同意。

**题项**	**1**	**2**	**3**	**4**	**5**
32. 我能够在需要时主动联系家庭医生进行医疗咨询。					
33. 当出现非紧急健康问题时，我首先向家庭医生寻求医疗服务。					

3 Psychometric results

This appendix provides the detailed psychometric results supporting the scale reliability, factor analysis, and construct validity assessment reported in the main manuscript.

Table S1. Reliability by scale dimension

Scale/Dimension	Items	N complete	Mean score	SD score	Cronbach alpha	Standardized alpha	Mean inter item r
Geographic Accessibility	1	4578	0.529	0.499			
Financial Affordability	1	4578	3.538	1.137			
Availability	3	4578	3.397	1.084	0.909	0.909	0.769
Acceptability	3	4578	3.364	0.945	0.861	0.861	0.674
Accommodation	3	4578	3.359	0.952	0.836	0.837	0.632
Accountability	3	4578	3.496	1.071	0.762	0.762	0.516
Perceived Feasibility	4	4578	3.471	0.896	0.884	0.885	0.658
Perceived Desirability	4	4578	3.379	0.952	0.917	0.917	0.735
Perceived Continuity	3	4578	3.493	0.869	0.856	0.857	0.667

Table S2. Item-total statistics for multi-item dimensions

Dimension	Corrected item total r	Alpha if deleted	Std alpha if deleted
Availability	0.849	0.843	0.843
Availability	0.775	0.904	0.905
Availability	0.832	0.857	0.859
Acceptability	0.803	0.741	0.741
Acceptability	0.735	0.806	0.806
Acceptability	0.675	0.861	0.861
Accommodation	0.652	0.818	0.819
Accommodation	0.759	0.714	0.715
Accommodation	0.685	0.784	0.786
Accountability	0.592	0.683	0.684

Accountability	0.650	0.615	0.616
Accountability	0.542	0.737	0.737
Perceived Feasibility	0.730	0.857	0.859
Perceived Feasibility	0.767	0.843	0.845
Perceived Feasibility	0.718	0.862	0.864
Perceived Feasibility	0.776	0.840	0.841
Perceived Desirability	0.779	0.903	0.904
Perceived Desirability	0.874	0.870	0.871
Perceived Desirability	0.783	0.902	0.902
Perceived Desirability	0.808	0.894	0.894
Perceived Continuity	0.669	0.853	0.855
Perceived Continuity	0.800	0.733	0.733
Perceived Continuity	0.721	0.805	0.806

Table S3. Primary rotated factor loadings

Dimension	Primary factor	Primary loading
Availability	Factor 2	0.882
Availability	Factor 2	0.818
Availability	Factor 2	0.859
Acceptability	Factor 3	0.845
Acceptability	Factor 3	0.825
Acceptability	Factor 3	0.708
Accommodation	Factor 4	0.764
Accommodation	Factor 4	0.806
Accommodation	Factor 4	0.730
Accountability	Factor 5	0.844
Accountability	Factor 5	0.749
Accountability	Factor 6	0.800
Perceived Feasibility	Factor 1	0.834
Perceived Feasibility	Factor 1	0.823

Perceived Feasibility	Factor 1	0.767
Perceived Feasibility	Factor 1	0.814
Perceived Desirability	Factor 1	0.767
Perceived Desirability	Factor 1	0.820
Perceived Desirability	Factor 1	0.765
Perceived Desirability	Factor 1	0.783
Perceived Continuity	Factor 1	0.776
Perceived Continuity	Factor 1	0.836
Perceived Continuity	Factor 1	0.791

Table S4. Inter-dimension correlation matrix

Dimension	Geographic Accessibility	Financial Affordability	Availability	Acceptability	Accommodation	Accountability	Perceived Feasibility	Perceived Desirability	Perceived Continuity
Geographic Accessibility	1.000	-0.017	-0.024	0.024	0.007	-0.050	0.169	0.120	0.162
Financial Affordability	-0.017	1.000	-0.065	-0.090	-0.029	-0.026	-0.004	0.022	0.016
Availability	-0.024	-0.065	1.000	0.542	0.574	0.446	0.287	0.343	0.315
Acceptability	0.024	-0.090	0.542	1.000	0.554	0.509	0.333	0.359	0.348
Accommodation	0.007	-0.029	0.574	0.554	1.000	0.506	0.319	0.337	0.358
Accountability	-0.050	-0.026	0.446	0.509	0.506	1.000	0.296	0.293	0.284
Perceived Feasibility	0.169	-0.004	0.287	0.333	0.319	0.296	1.000	0.809	0.841
Perceived Desirability	0.120	0.022	0.343	0.359	0.337	0.293	0.809	1.000	0.794
Perceived Continuity	0.162	0.016	0.315	0.348	0.358	0.284	0.841	0.794	1.000

Table S5. Initial eigenvalues from factor analysis

Component	Eigenvalue
Component 1	9.685
Component 2	3.779
Component 3	1.287

Component 4	1.071
Component 5	0.996
Component 6	0.654
Component 7	0.598
Component 8	0.509
Component 9	0.449
Component 10	0.427
Component 11	0.412
Component 12	0.383
Component 13	0.381
Component 14	0.349
Component 15	0.317
Component 16	0.278
Component 17	0.254
Component 18	0.239
Component 19	0.225
Component 20	0.216
Component 21	0.198
Component 22	0.163
Component 23	0.130

Note. Eigenvalues were calculated from the correlation matrix of the 23 multi-item scale variables before rotation. Components 1-23 represent the initial Components derived from these 23 items and do not indicate that 23 factors were retained.
